# Andrographolide in hematological malignancies: potential mechanisms and translational challenges

**DOI:** 10.3389/fphar.2026.1942161

**Published:** 2026-10-15

**Authors:** Tiantian Wang, Yulu Wang, Haiyun Liu, Sheng Yuan, Tingting Liu

**Affiliations:** 1 The Second Clinical Medical College, Jiangxi University of Chinese Medicine, Nanchang, Jiangxi, China; 2 Department of Hematology, Jiangxi Provincial People’s Hospital, The First Affiliated Hospital of Nanchang Medical College, Nanchang, Jiangxi, China; 3 Jiangxi Provincial Key Laboratory of Hematologic Diseases, Nanchang, Jiangxi, China; 4 Jiangxi Provincial Key Laboratory of Hematological Diseases, Department of Hematology, The First Affiliated Hospital of Nanchang University, Nanchang, Jiangxi, China; 5 Jiangxi Clinical Research Center for Hematologic Disease, Nanchang, Jiangxi, China; 6 Department of Clinical Laboratory, Jiangxi Provincial People’s Hospital, The First Affiliated Hospital of Nanchang Medical College, Nanchang, Jiangxi, China; 7 Department of Pathology, Jiangxi Provincial People’s Hospital, The First Affiliated Hospital of Nanchang Medical College, Nanchang, China

**Keywords:** andrographolide, apoptosis, ferroptosis, hematological malignancies, inflammatory signaling

## Abstract

Hematological malignancies comprise a heterogeneous group of life-threatening diseases arising from the hematopoietic system. Despite substantial advances in immunotherapies and molecularly targeted agents, drug resistance and disease relapse remain formidable clinical challenges. The natural product andrographolide, a diterpenoid lactone isolated from *Andrographis paniculata*, has attracted increasing research interest for its antitumor potential. This narrative review summarizes and discusses preclinical studies on andrographolide in hematological malignancies, covering its reported antitumor activities, drug synergy, and derivative development. Andrographolide has been reported to trigger intrinsic apoptosis through reactive oxygen species burst, while also suppressing STAT3 signaling via a ROS-dependent mechanism, thereby inducing tumor cell apoptosis. In addition, andrographolide has been reported to induce ferroptosis in a cell context-dependent manner, potentially through regulation of the p38/Nrf2/HO-1 pathway and upregulation of ACSL4 (also known as FACL4) expression; it also exerts anti-proliferative effects through metabolic reprogramming and induction of cellular differentiation. Andrographolide further suppresses inflammatory signaling in malignant cells through blockade of NF-κB signaling. In combination, andrographolide exhibits synergistic effects with cytarabine, vincristine, dexamethasone, and other agents in certain experimental settings. Finally, derivatives and analogs of andrographolide have also been explored.

## 1 Introduction

Hematological malignancies comprise a diverse group of neoplasms originating from the hematopoietic system, with common subtypes including leukemia, lymphoma, multiple myeloma, and myeloproliferative neoplasms. In recent years, the global incidence of these diseases has continued to rise, imposing an escalating disease burden worldwide (Zhang et al., 2023). Encouragingly, the emergence of innovative therapeutic modalities—particularly immunotherapies and molecularly targeted agents—has substantially improved clinical outcomes for affected patients (Tang et al., 2023; Huang et al., 2024). Among immunotherapeutic approaches, representative strategies include bispecific antibodies such as teclistamab, talquetamab, blinatumomab, and glofitamab (Verkleij et al., 2024; Lertvivatpong and Inaba, 2026; Zhou et al., 2025), as well as chimeric antigen receptor T-cell (CAR-T) therapies, which primarily target BCMA and CD19 (Rade et al., 2026; Deschênes-Simard et al., 2025). Molecularly targeted agents encompass Bcr-Abl tyrosine kinase inhibitors (e.g., imatinib, flumatinib) (Jabbour and Kantarjian, 2025), Bruton’s tyrosine kinase (BTK) inhibitors (e.g., ibrutinib, zanubrutinib) (Alu et al., 2022), Janus kinase (JAK) inhibitors (e.g., ruxolitinib, golidocitinib) (Tremblay et al., 2025; Song et al., 2024), as well as the anti-CD20 monoclonal antibody rituximab (Coiffier et al., 2002) and anti-CD38 monoclonal antibodies daratumumab and isatuximab (Facon et al., 2019; Dhillon, 2020). Despite these advances, a substantial subset of patients fails to derive sustained benefit from these novel therapies due to primary or acquired drug resistance, rapid disease relapse, and off-target toxicities (Ruella et al., 2023; Shyam Sunder et al., 2023; de Assis et al., 2023). Therefore, the exploration of novel therapeutic strategies remains of paramount importance for improving clinical outcomes in these patients.

Natural products occupy a pivotal position in anticancer drug discovery by virtue of their structural diversity and distinctive bioactivities. The clinical value of artemisinin in the treatment of human diseases has long been internationally recognized (Miller and Su, 2011; Tu, 2016), and its success exemplifies the unique advantages of natural products in pharmaceutical development. In the context of hematological malignancies, natural products have likewise demonstrated considerable antitumor potential. Several natural product-derived agents are currently utilized in clinical practice for hematological malignancies—for instance, homoharringtonine for the treatment of acute myeloid leukemia (AML) (Jin et al., 2013), and arsenic trioxide as a first-line therapeutic agent for acute promyelocytic leukemia (Burnett et al., 2015)—both serving as representative examples of natural product-derived anti-hematological malignancy therapeutics. Concurrently, the antitumor potential of numerous additional natural products is being actively investigated, with metabolites such as celastrol, curcumin, resveratrol, and epigallocatechin gallate (EGCG) all demonstrating anti-hematological malignancy activity in preclinical studies (Zhong et al., 2019; Kouhp et al., 2019; Espinoza et al., 2019; Della Via et al., 2021). Recent studies on natural products across various cancer types have further demonstrated that their mechanisms of action and therapeutic outcomes vary considerably according to the specific disease context (Li D. et al., 2024; Facai et al., 2025). Among the various classes of natural products, diterpenoid compounds have shown particularly prominent potential (Zeng et al., 2012; Sh et al., 2020; Sousa-Pimenta et al., 2023), and within this chemical class, andrographolide has recently become a growing research focus (Figure 1).

**FIGURE 1:**
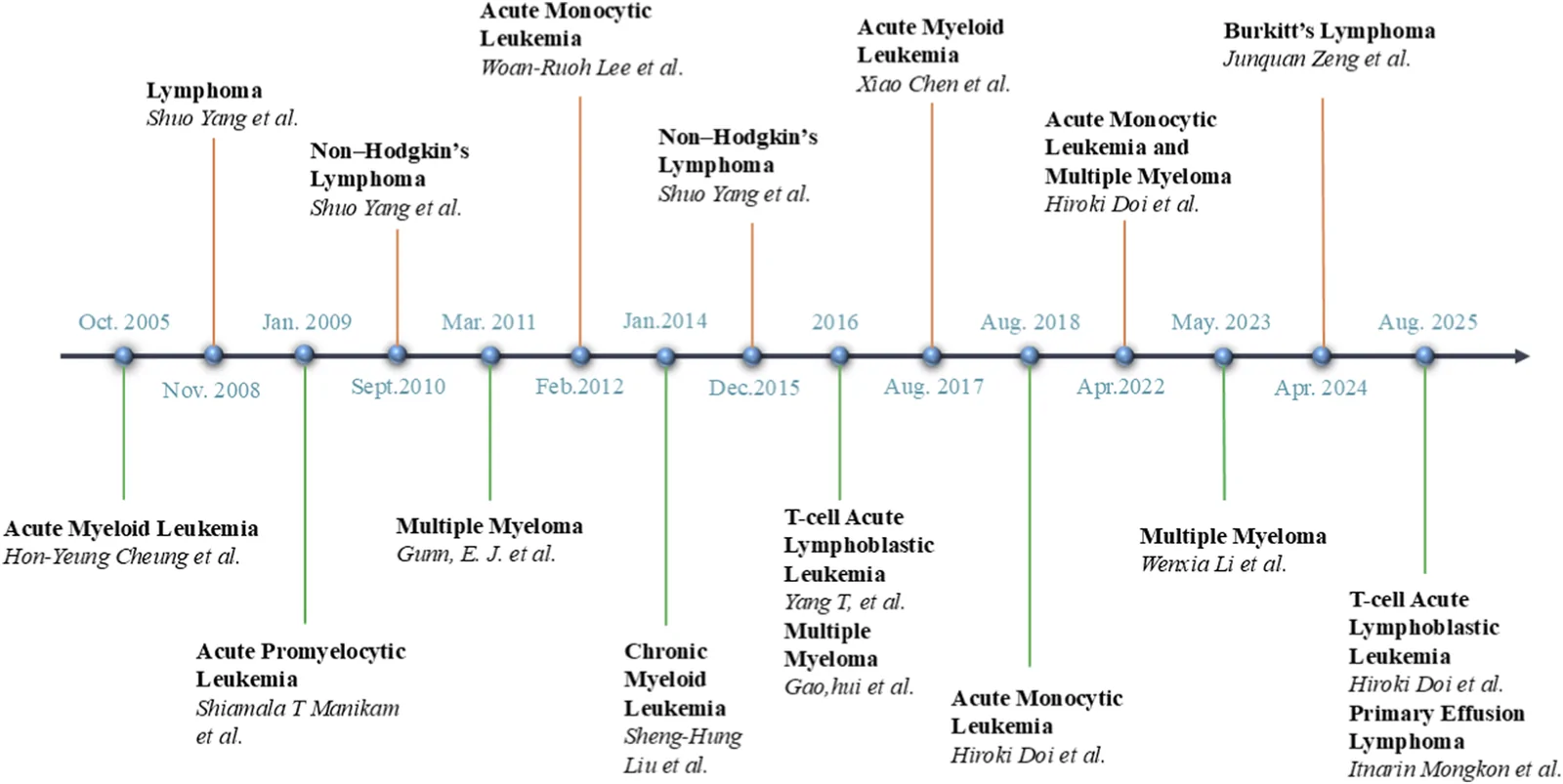
Timeline of key discoveries and research progress of andrographolide (AG) in hematological malignancies. Chronological summary of major research milestones in AG for hematological malignancies. The figure and all its graphical elements are original. The data and events summarized are derived from the primary literature cited in the main text.

Andrographolide (AG), the principal active metabolite of the botanical drug *Andrographis paniculata*, which is widely used in traditional Asian medicine, exhibits multiple pharmacological activities, including anti-inflammatory (Liu et al., 2021), antiviral (Murugan et al., 2021), antitumor (Wang et al., 2025), antioxidant (Gan et al., 2019), and immunomodulatory effects (Wang et al., 2022). Clinical research on AG in hematological malignancies remains limited. In recent years, however, the antitumor activity of AG has been increasingly reported in preclinical models. A recent pan-cancer review summarized these findings but focused mainly on solid tumors and discussed hematological malignancies only briefly (Hu et al., 2024). Therefore, the preclinical evidence for AG in hematological malignancies remains insufficiently synthesized. To this end, this review focuses on preclinical studies of AG in hematological malignancies, summarizes the current findings, and outlines future research directions.

### 1.1 Literature search strategy

A literature search was performed in PubMed and Web of Science up to June 2026 using combinations of the following keywords: “andrographolide”, “Andrographis paniculata”, “hematological malignancies”, “leukemia”, “lymphoma”, “multiple myeloma”, “myeloproliferative neoplasms”, “apoptosis”, and “ferroptosis”. Boolean operators (AND, OR) were used to combine the search terms. Full-length articles and other relevant records, including conference abstracts, were included. Non-English articles and case reports were excluded. Additional articles were identified by manually screening the reference lists of the retrieved articles.

## 2 The anti-tumor mechanisms of AG

The molecular mechanisms through which AG exerts its antitumor effects in hematological malignancies are complex and involve the coordinated regulation of multiple biological processes, including apoptosis, ferroptosis, cell cycle arrest, differentiation, and inflammatory signaling.

### 2.1 Apoptosis

Apoptosis is a tightly regulated, genetically programmed form of cell death, characterized by loss of mitochondrial membrane potential, activation of the caspase family, and PARP cleavage. This process plays a critical role in both tumor therapy and the development of drug resistance (Carneiro and El-Deiry, 2020). Multiple studies have reported that AG exhibits dose-dependent pro-apoptotic activity across various hematological malignancy cell lines (Doi et al., 2018; Doi et al., 2017), inducing apoptosis through distinct mechanisms depending on cellular context. The primary molecular pathways are detailed below (Figure 2).

**FIGURE 2:**
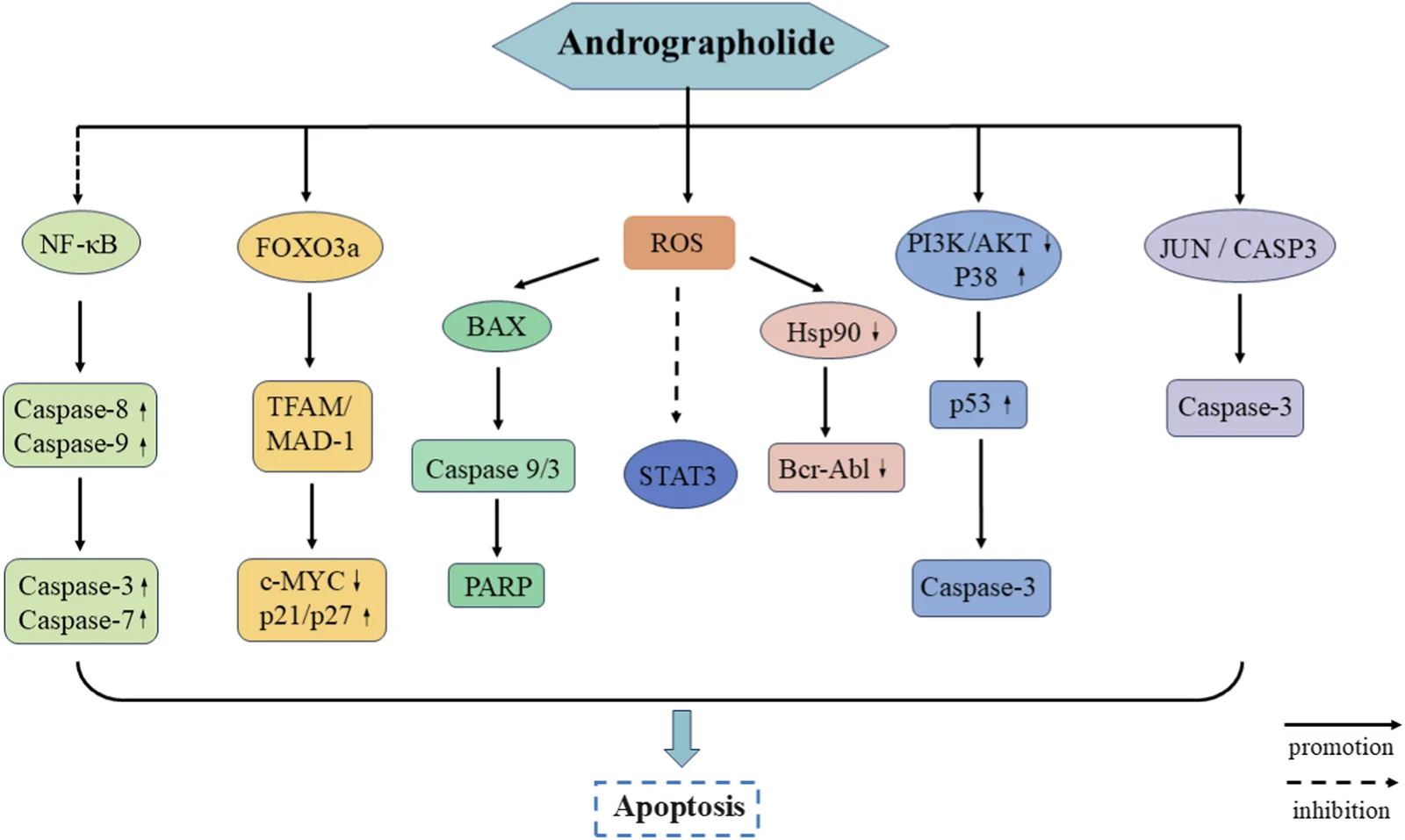
Mechanisms of AG-induced apoptosis and associated signaling pathways. Schematic diagram of the apoptotic signaling pathways modulated by AG in hematological malignancy cells. All graphical elements are original.

#### 2.1.1 ROS-dependent and mitochondria-mediated apoptosis

A well-characterized mechanism by which AG induces apoptosis in hematological malignancy cells centers on the intrinsic mitochondrial apoptotic pathway triggered by reactive oxygen species (ROS) burst. However, the activation efficiency and coordinated regulation of this pathway exhibit distinct cell type-specific response characteristics across different lineages and genetic backgrounds.

In lymphoma cells—including Burkitt lymphoma, mantle cell lymphoma, follicular lymphoma, and diffuse large B-cell lymphoma—AG (IC_50_ 15–40 μM at 48 h) treatment markedly elevates ROS levels, subsequently inducing conformational changes in BAX, triggering mitochondrial membrane potential (ΔΨm) collapse, and ultimately activating caspase-9/3 with consequent PARP cleavage (Yang et al., 2010). In HL-60 AML cells, AG-induced mitochondrial cytochrome c release and an increased BAX/Bcl-2 ratio have similarly been observed (Hon-Yeung et al., 2005). Likewise, in THP-1 monocytic leukemia cells, ROS-dependent mitochondrial depolarization and caspase-3/7 activation have been confirmed (Doi et al., 2022). The critical initiating role of upstream ROS has been further validated through pharmacological intervention: the antioxidant N-acetylcysteine (NAC) substantially abrogates the aforementioned apoptotic effects, whereas the glutathione-depleting agent buthionine sulfoximine (BSO) effectively enhances AG’s cytotoxic activity (Yang et al., 2010).

Notably, despite the high degree of conservation of the core pathway, significant heterogeneity exists in the sensitivity and depth of response among different hematological malignancy cells, suggesting the involvement of cell type-specific modulatory factors. A paradigmatic example is the H929 multiple myeloma cell line, which exhibits marked sensitivity to AG yet displays comparatively low sensitivity to the conventional chemotherapeutic agent cytarabine. This observation may suggest a mechanism distinct from classical DNA damage responses, but direct evidence is lacking (Doi et al., 2022).

#### 2.1.2 Regulation of the STAT3 signaling pathway

AG can also disrupt pro-survival signaling networks on which tumor cells depend through ROS-dependent mechanisms, thereby enhancing its cytotoxic effects. In primary effusion lymphoma (PEL) cells, AG time-dependently suppresses STAT3 phosphorylation at Tyr705, without affecting the phosphorylation levels of NF-κB p65 or AKT, indicating selective inhibition of the STAT3 pathway (Mongkon et al., 2025). This STAT3 inhibition operates in parallel with mitochondrial apoptosis, as both events are equally dependent on upstream ROS burst (Mongkon et al., 2025). The *in vivo* efficacy of AG in PEL xenograft models (500 mg/kg/day, oral) has been validated, with significant tumor reduction and no observable toxicity (Mongkon et al., 2025); however, this mechanism has only been demonstrated in PEL cells, and its generalizability to other subtypes remains untested.

#### 2.1.3 Regulation of the PI3K/AKT and p38 MAPK pathways

In Jurkat cells, a T-cell acute lymphoblastic leukemia (T-ALL) cell line, AG (IC_50_ 26.5 μM at 48 h) treatment coordinately suppresses the PI3K/AKT survival pathway while activating the p38 MAPK stress response pathway: it reduces AKT phosphorylation to relieve pro-survival signaling while concurrently enhancing p38 MAPK phosphorylation to initiate stress-responsive signaling, accompanied by upregulated p53 phosphorylation. Following this signaling remodeling, AG induces caspase-3-dependent apoptosis in Jurkat cells. The *in vivo* relevance of the caspase-3-dependent apoptotic pathway in Jurkat cells has been corroborated in xenograft models, where AG (5–20 mg/kg) dose-dependently inhibited tumor growth (Yang et al., 2016).

Interestingly, whether ROS is required for AG-induced apoptosis in Jurkat cells remains controversial. An initial report indicated that AG (10 μg/mL, ≈28.5 μM, 48 h) induces apoptosis without detectable ROS generation, suggesting that AG can bypass the canonical ROS–mitochondria axis and directly engage the PI3K/AKT and p38 MAPK pathways (Yang et al., 2016). Conversely, a subsequent study (50 μM, ≈17.5 μg/mL, 24 h) demonstrated ROS production and mitochondrial membrane depolarization, supporting a ROS–mitochondria-dependent apoptotic mechanism (Doi et al., 2024; Doi et al., 2025). These discrepancies may be attributable to differences in drug concentration, treatment duration, and ROS detection assays. Therefore, future studies should evaluate AG-induced ROS generation, mitochondrial depolarization, and apoptosis under identical experimental conditions to determine whether these findings are reproducible and to clarify the influence of specific experimental parameters.

#### 2.1.4 Regulation of JUN and CASP3

In Burkitt lymphoma, AG exerts its inhibitory effects by putatively targeting JUN and CASP3. Network pharmacology predictions and molecular docking analyses suggested these two proteins as potential interacting candidates. Subsequent *in vitro* experiments (IC_50_ 70–136 μM) indicated that AG upregulates JUN and CASP3 protein expression, thereby inhibiting Burkitt lymphoma proliferation and inducing apoptosis (Zeng et al., 2024). *In vivo* efficacy was validated in patient-derived xenograft (PDX) models (25 mg/kg, i. p.), with considerable tumor suppression (Zeng et al., 2024).

#### 2.1.5 Additional apoptosis-related mechanisms

The molecular mechanisms through which AG induces apoptosis can be further expanded from the following perspectives. First, AG promotes FOXO3a nuclear translocation, which subsequently downregulates *c-MYC* expression through mitochondrial proteins (TFAM and MAD-1) and upregulates the cyclin-dependent kinase inhibitors p21 and p27, concomitantly causing loss of mitochondrial membrane potential and ultimately inducing apoptosis in lymphoma cells. FOXO3a gene silencing abrogates these effects, confirming that FOXO3a serves as a critical mediator of this pathway (Yang et al., 2015). Second, AG exhibits cytotoxicity against multiple myeloma cancer stem cells (MM-CSCs), with a reported LC_50_ of 1.5–3.5 μM (Gunn et al., 2011). As a known NF-κB inhibitor, AG simultaneously activates initiator caspase-8 (extrinsic pathway) and caspase-9 (intrinsic pathway) in MM-CSCs, converging on effector caspase-3/7 cascade activation and inducing MM-CSC apoptosis (Gunn et al., 2011; Gao and Wang, 2016). Furthermore, in CML K562 cells, AG interferes with Hsp90 chaperone function, promotes the degradation of client proteins such as Bcr-Abl through ROS-dependent cleavage of Hsp90α, induces apoptosis, and suppresses oncoprotein-driven malignant transformation (Liu et al., 2014).

#### 2.1.6 Negative regulation of apoptosis by protective autophagy

Concurrent with the initiation of apoptotic signaling, AG also triggers protective autophagy, constituting an intrinsic negative feedback buffering mechanism. In lymphoma cells, pre-treatment with the antioxidant NAC blocks both autophagy and apoptosis, suggesting that autophagy initiation depends on AG-induced ROS production (Yang et al., 2008). These findings reveal the existence of an endogenous negative feedback loop within AG’s mechanism of action, and suggest that combined intervention with autophagy inhibitors and AG may theoretically possess synergistic sensitization potential. Whether similar protective responses occur in other hematological malignancies remains unknown, as this effect has only been characterized in lymphoma models.

### 2.2 Ferroptosis

Ferroptosis is a non-apoptotic form of cell death driven by iron-dependent accumulation of lipid peroxidation, characterized by intracellular free iron accumulation, glutathione depletion, GPX4 inactivation, and mitochondrial shrinkage (Wahida and Conrad, 2025; Liu et al., 2025). Recent studies have suggested that AG may trigger ferroptosis in certain hematological malignancy models.

The mechanisms by which AG induces ferroptosis involve multiple signaling pathways across different hematological malignancies (Figure 3), although the strength of evidence varies across different experimental models. In T-ALL and B-cell acute lymphoblastic leukemia (B-ALL) cells, AG treatment results in a sharp elevation of intracellular ROS levels, which in turn induces upregulation of the ferroptosis-associated marker ACSL4, accompanied by characteristic mitochondrial damage, including reduction in mitochondrial area and loss of cristae architecture (Doi et al., 2024; Doi et al., 2025). However, these studies did not use rescue experiments to further confirm ferroptosis. Therefore, these findings are insufficient to demonstrate ferroptotic cell death in these leukemia cells. By comparison, in human multiple myeloma cells, the induction of ferroptosis by AG (20–40 μM) through the p38/Nrf2/HO-1 pathway is supported by elevated intracellular and mitochondrial Fe^2+^ levels, increased lipid peroxidation, reduced GPX4 and SLC7A11 expression, and rescue by ferroptosis-specific inhibitors Ferrostatin-1 (Fer-1) and deferoxamine (DFOM) (Li et al., 2023).

**FIGURE 3:**
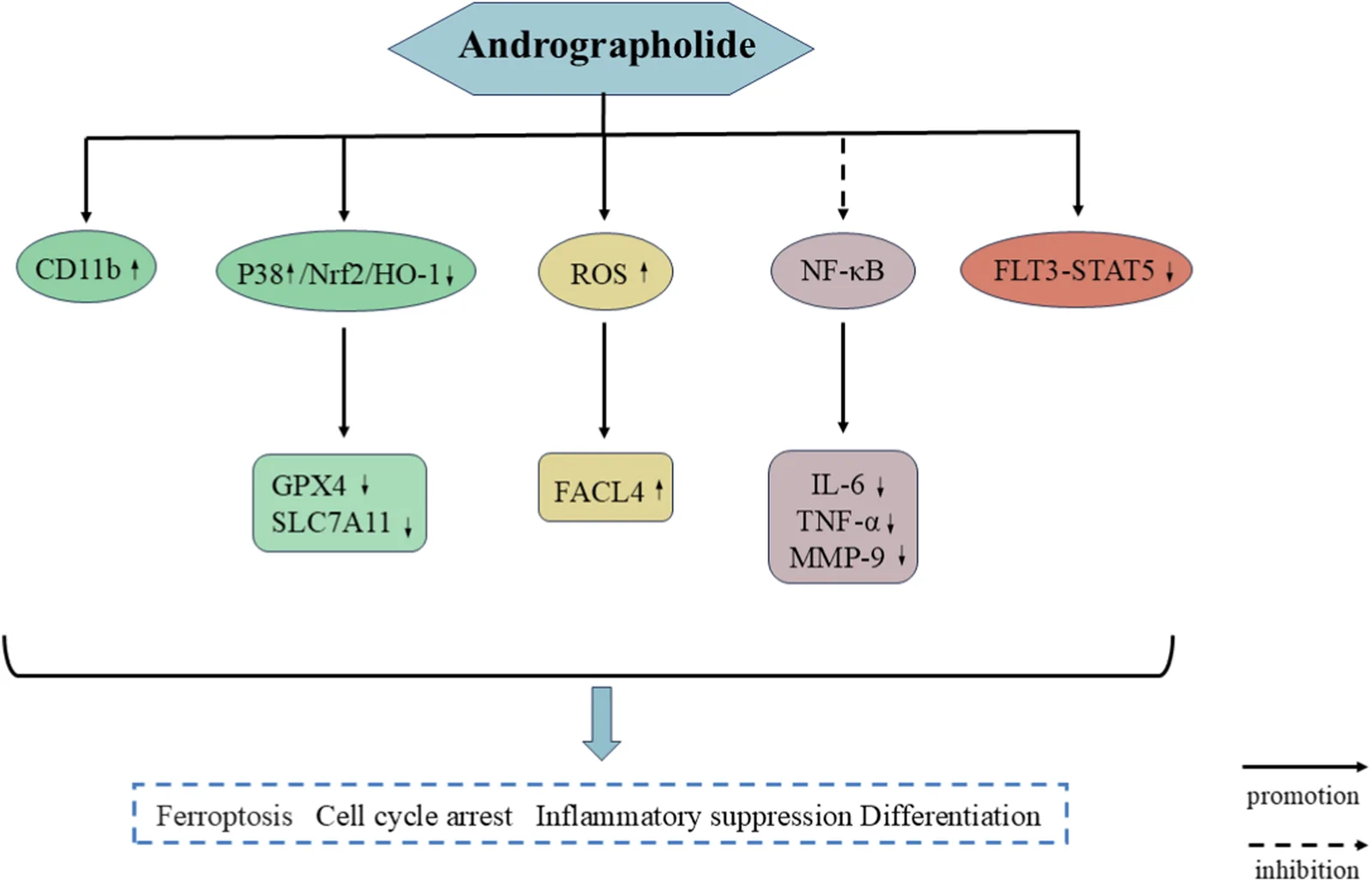
Mechanisms of AG-induced ferroptosis, cell cycle arrest, differentiation, and inflammatory suppression. Schematic summarizing the key signaling pathways through which AG modulates ferroptosis, cell cycle progression, differentiation, and inflammatory signaling in hematological malignancy cells. All graphical elements are original.

### 2.3 Cell cycle arrest and differentiation regulation

Cell cycle arrest refers to the halting of cells at specific checkpoints, preventing progression to the next division phase and thereby suppressing proliferation (Matthews et al., 2022). Cellular differentiation denotes the process by which cells transition from an immature state toward a mature state with specialized function and morphology (Ntziachristos et al., 2016). AG exerts regulatory effects on both of these biological processes (Figure 3), inhibiting the malignant proliferation of tumor cells through distinct yet complementary mechanisms.

In terms of cell cycle regulation, AG treatment has been shown to induce G_0_/G_1_ phase arrest in HL-60 AML cells (Hon-Yeung et al., 2005). However, studies in T-ALL cells revealed no alteration in cell cycle distribution following AG treatment (Doi et al., 2025), suggesting that the effects of AG on cell cycle progression are cell type-specific. Furthermore, AG inhibits MV4-11 cell proliferation by downregulating *FASN* (fatty acid synthase)/*ACACA* (acetyl-CoA carboxylase alpha) to suppress fatty acid synthesis and by blocking the FLT3-STAT5 signaling axis to inhibit protein synthesis (Chen et al., 2017). Although this mechanism does not directly involve classical cell cycle checkpoints, it may indirectly influence cell cycle progression at the metabolic level. In addition to cell cycle modulation, AG also induces cell differentiation. In NB4 acute promyelocytic leukemia cells, AG (IC_50_ 4.5 μM) elicits potent differentiation activity, as evidenced by the upregulation of the differentiation marker CD11b (Manikam and Stanslas, 2009).

### 2.4 Regulation of inflammatory signaling in malignant cells

In chronic inflammatory settings, persistent activation of the NF-κB signaling pathway promotes tumor cell proliferation, survival, and invasion, with inflammatory cytokines (IL-6, TNF-α) and matrix metalloproteinases (MMPs, particularly MMP-9) serving as key mediators (Zhao et al., 2021; Al-Kadash et al., 2026). AG suppresses inflammatory signaling in malignant cells through inhibition of NF-κB activation, with regulatory effects encompassing cytokine secretion and MMP-9 expression (Figure 3).

In THP-1 monocytic leukemia cells, AG significantly inhibits lipopolysaccharide-induced production of the inflammatory cytokines IL-6 and TNF-α, an effect closely associated with suppression of NF-κB signaling (Doi et al., 2018). In the same cell line, another study further demonstrated that AG concentration-dependently inhibits MMP-9 activation, protein expression, and mRNA transcription upon TNF-α or LPS stimulation, with the underlying mechanism involving inhibition of IκB-α degradation and blockade of NF-κB nuclear translocation and transcriptional activity (Woan-Ruoh et al., 2012). These findings indicate that AG, through targeting the core nodal points of the NF-κB pathway in THP-1 cells, simultaneously suppresses both inflammatory responses and the expression of invasion-associated proteins.

### 2.5 Combined application

Several studies have reported that AG can produce synergistic effects when combined with certain antineoplastic agents, although the outcome is not universally consistent across all cell types and drug combinations (Figure 4). Synergy was defined by the Chou–Talalay combination index (CI) method, CI < 1, CI = 1, and CI > 1 indicate synergy, additivity, and antagonism, respectively (Chou, 2010). AG primarily induces tumor cell apoptosis through ROS-dependent mitochondrial dysfunction, a mechanism that differs substantially from conventional cell cycle-specific agents such as cytarabine or vincristine, rendering it a mechanistically rational candidate for combined application based on mechanistic complementarity (Doi et al., 2025). In U937 AML cells, AG pretreatment (10 μM, 24 h) followed by topotecan (0.3 μM) markedly potentiated antitumor activity, as evidenced by enhanced S-phase arrest and increased apoptosis (Hodroj et al., 2018; Rizk et al., 2016). In CEM-C1 glucocorticoid-resistant acute lymphoblastic leukemia cells, AG (5 μM) combined with dexamethasone (50 μM) at a fixed ratio of 1:10 for 24 h synergistically suppressed cell growth (CI < 1), with an inhibition rate of 51%; mechanistically, this combination activated the PI3K/AKT/mTOR pathway and inhibited autophagy, thereby upregulating glucocorticoid receptor expression and restoring dexamethasone sensitivity (Li X. et al., 2024). In multiple myeloma (H929) and plasma cell leukemia (ARH77) cells, low-dose AG (3 μM) combined with graded concentrations of Ara-C (12–40 μM) or vincristine (0.03–0.1 μM) for 24 or 48 h exhibited synergistic effects (CI < 1), increasing the apoptotic fraction by approximately 2-fold compared with AG alone (Doi et al., 2026). In these combination models, AG’s effect was not primarily cell-cycle-dependent; this contrasts with the cell cycle-dependent action of these conventional agents and may provide functional complementarity with cycle-specific drugs. Importantly, this synergistic effect may be cell type- and time-dependent, as antagonism (CI > 1) was observed in ARH77 cells at 48 h with certain AG + Ara-C combinations, despite synergy at 24 h (Doi et al., 2026). These findings are primarily derived from *in vitro* models and do not support broad clinical combination claims; further validation in animal models and clinical studies is required before future clinical translation can be considered.

**FIGURE 4:**
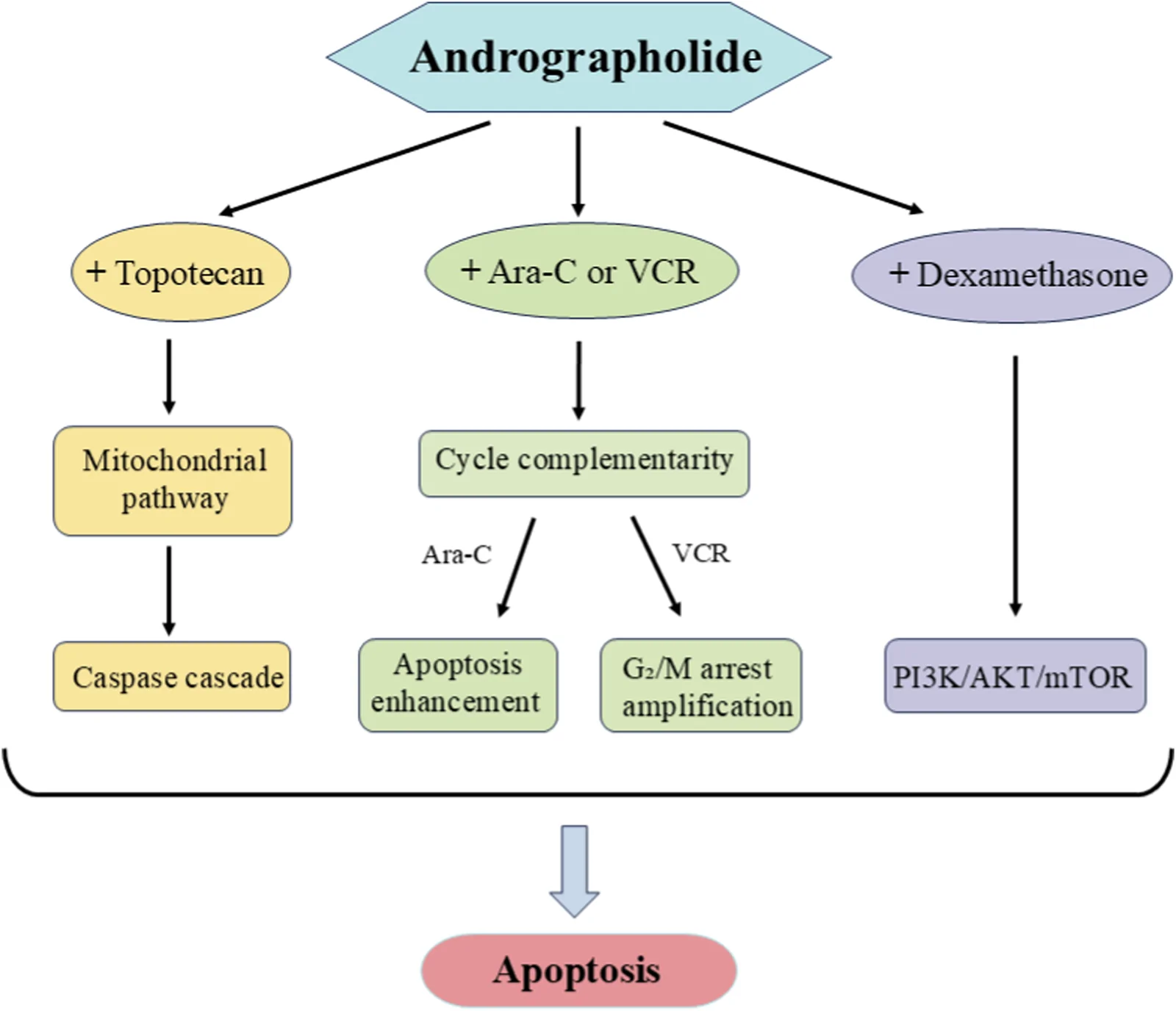
Synergistic mechanisms of AG in combination with conventional chemotherapeutic agents. Schematic illustrating the reported synergistic interactions and molecular mechanisms of AG combined with standard chemotherapeutic drugs in hematological malignancies. All graphical elements are original.

## 3 Andrographolide derivatives

Notably, although AG possesses substantial preclinical activity against hematological malignancies, its poor aqueous solubility, low oral bioavailability, and rapid *in vivo* metabolism severely constrain its future clinical translation (Loureiro Damasceno et al., 2022; Mehta et al., 2021). To overcome these limitations and develop more potent AG-based agents, multiple research groups have explored various structural derivatives using AG as a lead compound, and have reported their activities in hematological malignancies—with some derivatives demonstrating improved potency or novel mechanisms. However, structural modification alone does not guarantee improved pharmacokinetics or druggability, and such properties require systematic evaluation in future studies. Below, we summarize the current categories of AG analogs and derivatives and their proposed mechanisms of action (Figure 5); Table 1 summarizes the anti-proliferative activity and reported mechanisms of the AG derivatives in hematological malignancy cell lines.

**FIGURE 5:**
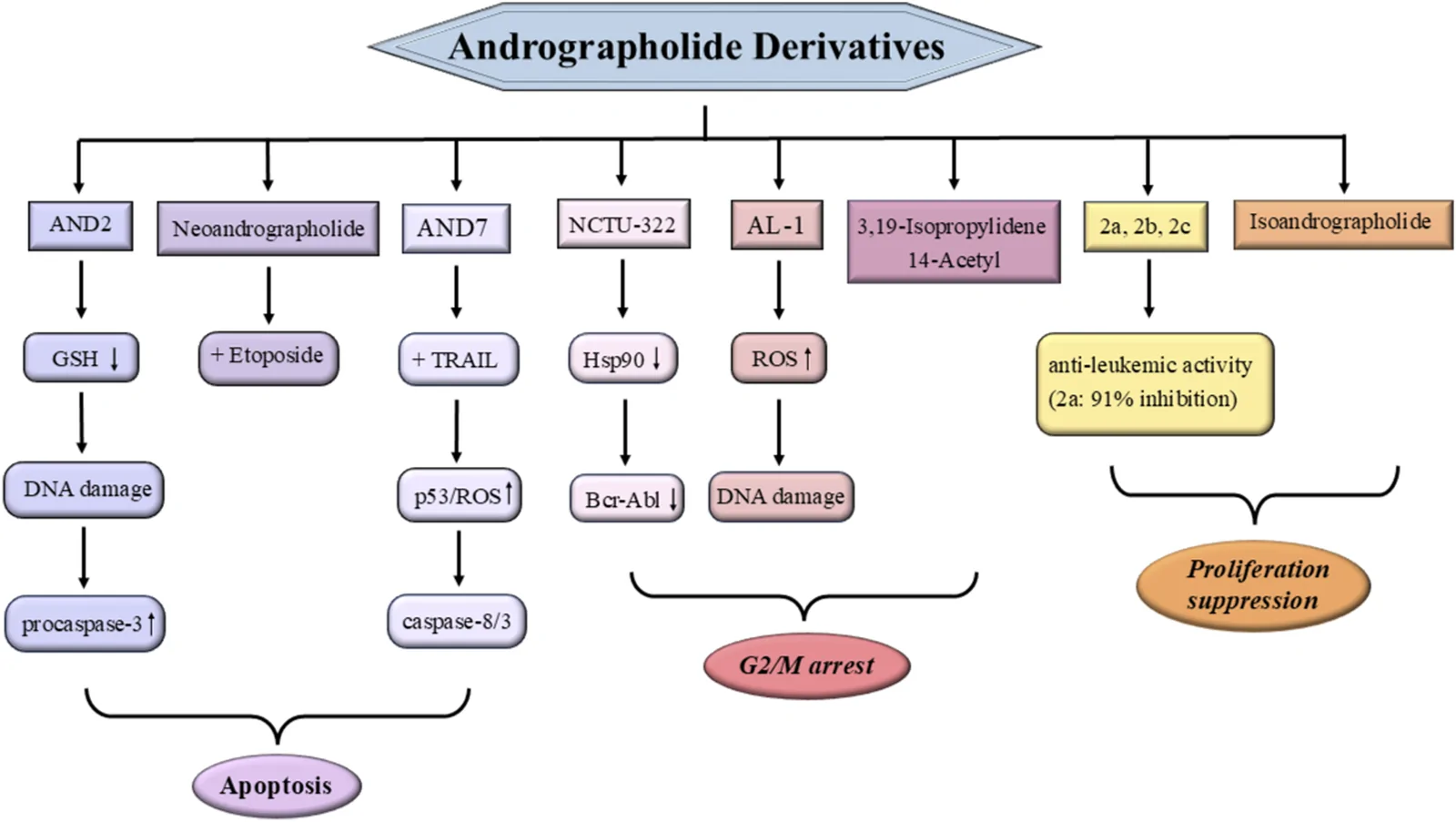
Representative AG derivatives and their reported mechanisms in hematological malignancy models. Schematic illustrating the reported mechanisms of action of selected AG derivatives against hematological malignancy cells. All graphical elements are original. Most derivatives were validated *in vitro* across multiple hematological malignancy cell lines; NCTU-322 was validated *in vitro* across multiple hematological malignancy cell lines and further confirmed in a KBM5R xenograft in nude mice (5 mg/kg i. p. every other day), where it significantly inhibited tumor growth. Evidence levels were assigned as Level 1 (*in vitro* + *in vivo*) for NCTU-322 and Level 2 (*in vitro* only) for the remaining derivatives.

**TABLE 1:** Anti-proliferative activity of andrographolide derivatives in hematological malignancy cell lines.

Derivative	Cell line/model	Potency			Selectivity	Mechanism	Ref.
		Andrographolide		Derivative			
Isoandrographolide	*In vitro* HL-60	GI_50_	9.33 μM	6.30 μM	-	Induces differentiation	(Chen et al., 2008)
Neoandrographolide	*In vitro* S-Jurkat	IC_90_	2.9 μM	20.8 μM	-	Chemosensitizer; enhances etoposide-induced apoptosis	(Pfisterer et al., 2010)
AND2	*In vitro* THP-1	IC_50_	-	35.21 μg/mL	Selective for leukemia cells	Redox-mediated cell death (GSH depletion)	(Raghavan et al., 2014)
AL-1	*In vitro* K562	IC_50_	30 μM	10 μM	Selective for leukemia cells	ROS-dependent apoptosis; G2/M arrest	(Zhu et al., 2013)
NCTU-322	*In vitro* K562, KBM5, KBM5R, K562R, THP-1, PBMC	IC_50_	23.66, 8.50, -, -, -, - μM	9.4, 3.46, 3.61, 26.95, >30, 10.9 μM	SI ≈ 3 (PBMC vs. KBM5)	Hsp90-dependent Bcr-Abl degradation; G2/M arrest; apoptosis	(Liao et al., 2019)
	*In vivo* KBM5R xenograft in nude mice		-	*In vivo*: 5 mg/kg i.p. every other day, significantly inhibited tumor growth			
AND7	*In vitro* U937, THP-1, ALL patient PBMCs, healthy PBMCs	IC_50_	73.03, -, -, - μM	6.63, 18.85, 23.74, >100 μM	SI > 15 (healthy PBMC vs. U937)	p53-regulated ROS accumulation; DR5/caspase-8/3 activation	(Xu et al., 2024)
3,19-Isopropylidene-AG	*In vitro* NCI-60 leukemia panel	GI_50_	-	10.23 μM (mean)	Selective for leukemia cells	Non-specific cell cycle arrest	(Jada et al., 2007)
14-Acetyl-AG	*In vitro* NCI-60 leukemia panel	GI_50_	-	6.45 μM (mean)	Selective for leukemia cells	Cell cycle arrest	(Jada et al., 2007)
2a	*In vitro* CCRF-CEM, HL-60(TB), K-562, RPMI-8226, SR	PGI (48 h)	-	90.97, 51.12, 56.31, 71.77, 63.12%	Selective for leukemia cells	-	(Tamang et al., 2022)
2b	*In vitro* CCRF-CEM, HL-60(TB), K-562, RPMI-8226, SR	PGI (48 h)	-	89.84, -, 55.49, 82.88, 70.99%	Selective for leukemia cells	-	(Tamang et al., 2022)
2c	*In vitro* CCRF-CEM, HL-60(TB), K-562, RPMI-8226, SR	PGI (48 h)	-	86.21, -, 47.29, 58.08, 83.82%	Selective for leukemia cells	-	(Tamang et al., 2022)

Among naturally occurring AG analogs, isoandrographolide exhibits superior anti-proliferative activity relative to the parent compound in HL-60 leukemia cells (Chen et al., 2008). Neoandrographolide possesses a unique chemosensitizing effect: in S-Jurkat T-cell leukemia cells, including XIAP-overexpressing drug-resistant variants, its combination with sublethal concentrations of etoposide (500 nM) significantly enhances apoptosis, increasing the apoptotic rate from 3.8% to 64.0% (Pfisterer et al., 2010). Importantly, deglycosylation of neoandrographolide results in a complete loss of this sensitizing activity, indicating that the glucose moiety is critical for its chemosensitizing effect (Pfisterer et al., 2010). 14-Deoxy-11,12-didehydroandrographolide (AND2) selectively inhibits THP-1 and Jurkat leukemia cells through glutathione depletion, DNA fragmentation, and upregulation of procaspase-3, exerting redox-mediated cell death without evidence of classical caspase-3 activation (Raghavan et al., 2014).

Among semisynthetic derivatives, structural modifications have further enhanced anti-leukemic activity or conferred novel mechanisms of action. AL-1 (andrographolide-lipoic acid conjugate) induces DNA damage, G2/M phase arrest, and mitochondrial apoptosis through a ROS-dependent pathway, and is approximately threefold more potent than the parent compound against K562 cells (Zhu et al., 2013). NCTU-322, a triply acetylated andrographolide derivative, downregulates the Bcr-Abl oncoprotein through an Hsp90-dependent mechanism and exhibits stronger effects than andrographolide on Bcr-Abl downregulation, Hsp90 cleavage, and cytotoxicity in both imatinib-sensitive and imatinib-resistant (including T315I mutant) CML cells (Liao et al., 2019). In addition, NCTU-322 also induces differentiation toward monocytic and granulocytic lineages, mitotic arrest, and apoptosis, and suppresses tumor growth *in vivo* (5 mg/kg, i. p., every other day) (Liao et al., 2019). AND7, in combination with TNF-related apoptosis-inducing ligand (TRAIL), upregulates DR5 through p53-regulated ROS accumulation and activates the caspase-8/caspase-3 cascade, reversing TRAIL resistance and exhibiting synergistic anti-acute lymphoblastic leukemia effects (Xu et al., 2024). Moreover, AND7 is approximately 11-fold more potent than andrographolide against U937 cells, and demonstrates high *in vitro* selectivity for leukemia cells over healthy peripheral blood mononuclear cells (PBMCs), selectivity index (SI) > 15 (Xu et al., 2024). Furthermore, 3,19-isopropylideneandrographolide and 14-acetylandrographolide demonstrate selective inhibitory activity against multiple leukemia cell lines (Jada et al., 2007). The benzylidene acetal derivatives 2a-2c achieve potent growth inhibition against CCRF-CEM leukemia cells; notably, 2a is the only derivative among the tested compounds that is active against HL-60 (TB) cells (Tamang et al., 2022).

## 4 Evidence quality and limitations

Throughout this review, “AG” refers to the purified diterpenoid lactone isolated from *Andrographis paniculata*, either commercially sourced or laboratory-purified. The reviewed evidence is heterogeneous, comprising mechanistic studies in immortalized cell lines, a limited number of animal models, and no dedicated clinical trials. A hierarchy of evidence should therefore be applied: xenograft and PDX models provide supportive *in vivo* evidence but are limited by small sample sizes and species differences, whereas the vast majority of evidence derives from immortalized cell lines, with some studies using relatively high AG concentrations. Overall, the main limitations can be summarized as follows: evidence is predominantly *in vitro*; experimental conditions and endpoints varied considerably across studies (e.g., AG concentrations, treatment durations, administration routes, and outcome measures), limiting direct comparison; and systematic safety assessment of AG and its derivatives is lacking, with toxicity data limited to preliminary observations. No dedicated clinical trial has evaluated AG in hematological malignancies, and the clinical efficacy, pharmacokinetics, and safety of AG in these patients remain unestablished.

## 5 Summary and prospect

The global incidence of hematological malignancies continues to rise, imposing a substantial disease burden that poses a serious threat to human health. Although innovative therapies—particularly immunotherapies and molecularly targeted agents—have markedly improved clinical outcomes, drug resistance and disease relapse remain formidable challenges that severely constrain long-term survival benefits. There is consequently an urgent need for novel therapeutic strategies with mechanistically distinct actions and improved toxicity profiles. Natural products, by virtue of their structural diversity and distinctive bioactivities, hold considerable research value in this regard.

Collectively, preclinical studies indicate that the anti-hematological malignancy effects of AG involve the coordinated regulation of multiple biological processes. AG induces apoptosis through ROS-mediated mitochondrial dysfunction and has been reported to trigger ferroptosis via lipid peroxidation, though the upstream regulatory pathways appear to be cell type-dependent. Other reported mechanisms include STAT3 suppression, PI3K/AKT inhibition, p38 MAPK activation, JUN/CASP3 upregulation, FOXO3a-mediated signaling, Hsp90 cleavage, and protective autophagy. In addition, AG modulates cell cycle progression, promotes differentiation, and suppresses inflammatory signaling through NF-κB blockade. In combination with conventional chemotherapeutics, AG exhibits synergistic effects that complement cycle-specific agents. Concurrently, the ongoing development of AG derivatives offers a viable route for structural optimization.

Despite these preclinical findings, several key issues remain to be addressed before clinical translation can be considered. To begin with, the precise molecular targets of AG remain to be fully elucidated, and the molecular mechanisms underlying its antitumor activity warrant further investigation. Furthermore, the poor aqueous solubility and low oral bioavailability of AG remain major bottlenecks. Nanocarrier-based delivery systems, structural modification, and combination strategies may offer potential solutions. However, structural modification alone is not sufficient to establish improved pharmacokinetics or druggability; therefore, experimental validation is needed to evaluate its effects on solubility and oral bioavailability. In addition, systematic assessment of AG and its derivatives is needed to define their exposure, toxicity, and therapeutic window. Robust analytical tools are also required to support pharmacokinetic assessment and quality control of AG derivatives and formulations. For example, recent methodological advances such as bioanalytical techniques for nanocarrier pharmacokinetics and chromatographic-spectrometric methods for drug quality control have provided valuable tools for this purpose (Meng et al., 2025; Lindenmaier et al., 2025). Finally, the optimal combination of AG with current clinical chemotherapeutic drugs remains to be further explored, including administration timing, dose ratios, and potential beneficiary populations across hematological malignancy subtypes. By addressing the above issues, the transition of AG from preclinical investigation to early-phase clinical studies in hematological malignancies may be facilitated.
